# Genetic association between selected cytokine genes and glioblastoma in the Han Chinese population

**DOI:** 10.1186/1471-2407-13-236

**Published:** 2013-05-12

**Authors:** Tianbo Jin, Xiaolan Li, Jiayi Zhang, Hong Wang, Tingting Geng, Gang Li, Guodong Gao, Chao Chen

**Affiliations:** 1 School of Life Sciences, Northwest University, Xi’an 710069, China; 2 National Engineering Research Center for Miniaturized Detection Systems, Xi’an 710069, China; 3 Department of Neurosurgery, Tangdu hospital, the Fourth Military Medical University, Xi’an 710038, China; 4 Mailbox 386, #229 North Taibai Road, Xi’an 710069, Shaanxi, China

**Keywords:** Cytokine gene, Glioblastoma (GBM), Tag single nucleotide polymorphism (tSNP), Case–control study

## Abstract

**Background:**

Glioblastoma (GBM) is the most malignant brain tumor. Many abnormal secretion and expression of cytokines have been found in GBM, initially speculated that the occurrence of GBM may be involved in these abnormal secretion of cytokines. This study aims to detect the association of cytokine genes with GBM.

**Methods:**

We selected seven tag single nucleotide polymorphisms (tSNPs) in six cytokine genes, which previously reported to be associated with brain tumors, and analyzed their association with GBM in a Han Chinese population using χ^2^ test and genetic model analysis.

**Results:**

We found two risk tSNPs and one protective tSNP. By χ^2^ test, the rs1801275 in *IL-4R* showed an increased risk of GBM. In the genetic model analysis, the genotype “TC” of rs20541 in *IL-13* gene showed an increased risk of GBM in over-dominant model (OR = 2.00; 95% CI, 1.13-3.54, *p* = 0.015); the genotype “CT” of rs1800871 in the *IL-10* gene showed a decrease risk in the over-dominant model (OR = 0.57; 95% CI, 0.33 – 0.97; *p* = 0.037). The genotype “AG” of rs1801275 in the *IL-4R* gene showed an increase risk in over-dominant model (OR = 2.29; 95% CI, 1.20 - 4.35; *p* = 0.0081) We further analyzed whether the six cytokine genes have a different effect on the disease in gender specific population, and found that the allele “G” of rs2243248 in the *IL-4* gene showed a decrease risk of GBM in female (OR = 0.35, 95% CI, 0.13 - 0.94, *p* = 0.0032), but the allele “T” showed a decrease risk in male (OR = 0.30, 95% CI, 0.17 - 0.53, *p* = 0.0032).

**Conclusions:**

Our findings, combined with previously reported results, suggest that cytokine genes have potential role in GBM development, which may be useful to early prognostics for GBM in the Han Chinese population.

## Background

Glioblastoma (GBM) is one of the most malignant and deadly brain tumors, and occurs more commonly in adults, especially in males. According to the classification of World Health Organization, it is classified as the highest grade of IV. Although GBM has been researched for many years, the etiology of it remains unclear. It possibly arises from genetic and epigenetic alterations in normal astroglial cells [1], implying that the genetic factors play the mainly role in GBM genesis.

Cytokines play a significant role in cancer diagnosis, prognosis and therapy. Current studies suggest that the occurrence and development of tumors such as glioma, gastric cancer and breast cancer are associated with cytokine genes [2-5]. Many abnormal secretion and expression of cytokines have been found in GBM. To examine whether cytokine genes also contribute to risk of GBM, we selected seven tag single nucleotide polymorphisms (tSNPs) in six cytokine genes, which previously reported to be associated with glioma susceptibility [2,4,6-10], to perform the study in the Han Chinese population using a case–control study.

## Methods

### Study population

We recruited 72 cases newly diagnosed and histological confirmed to be GBM for the molecular epidemiology study at the department of Neurosurgery, Tangdu Hospital, affiliated with The Fourth Military Medical University in Xi’an city, China. None of them had suffered other cancer. We also selected 302 healthy unrelated individuals from the medical examination center at Tangdu Hospital. To ensure that the controls were cancer-free, we tested their presence of plasma carcinoembryonic antigen and alpha-fetoprotein. All subjects were Han Chinese, and they were selected according to detailed recruitment and exclusion criteria.

### Clinical data and demographic information

A standard epidemiological questionnaire was used to collect personal data through in-person interview, including residential region, age, smoking status, gender, alcohol use, ethnicity, education status and family history of cancer. We collected the cases information through consultation with treating physicians or medical chart review. All of the participants signed informed consent. After the interview, we collected 5 ml peripheral blood from each subject according to the study protocol approved by the Clinical Research Ethics of Northwest University.

### tSNP selection and genotyping

We selected seven tSNPs from six cytokine genes, which previously published to be associated with brain tumors, with minor allele frequency (MAF) > 5% in the HapMap CHB (Chinese Han Beijing) population. DNA was extracted from whole-blood samples using GoldMag-Mini Whole Blood Genomic DNA Purification Kit according to the manufacture’s protocol (GoldMag Co. Ltd. Xian, China) and concentration was measured by spectrometry (DU530UV/VIS spectrophotometer, Beckman Instruments, Fullerton, CA, USA). We used Sequenom MassARRAY Assay Design 3.0 Software to design Multiplexed SNP MassEXTENDED assay [11]. SNP genotyping was performed by Sequenom MassARRAY RS1000 using the standard protocol. Data management and analysis was performed by Sequenom Typer 4.0 Software [11,12].

### Statistical analysis

Genotypic frequencies in controls for each tSNP were tested for departure from Hardy-Weinberg Equilibrium (HWE) using an exact test. A *p* = 0.05 was considered the threshold of statistical significance. We compared the allele frequencies of cases and controls using the χ^2^ test [13]. Odds Ratio (ORs) and 95% Confidence intervals (95% CIs) were calculated by unconditional logistic regression analysis adjusted for age and gender [14]. The most common genotype in the controls was used as reference group. The possibility of gender differences as a resource of population substructure was evaluated by a genotype test for each tSNP in males and females separately.

Statistical analyses were calculated by Microsoft Excel and SPSS 16.0 statistical package (SPSS, Chicago, IL). The associations between the cytokine genes and the risk of GBM were tested using genetic models (co-dominant, dominant, recessive, over-dominant and log-additive) analysis by SNP stats, website software from http://bioinfo.iconcologia.net/snpstats/start.htm. ORs and 95% CIs were calculated by unconditional logistic regression analysis adjusted for age and gender [14]. Akaike’s Information Criterion (AIC) and Bayesian Information Criterion (BIC) were used to determine the best-fitting model for each SNP.

## Results

A multiplexed SNP MassEXTEND assay was designed with the Sequenom MassARRAY Design 3.0 Software. Seven tSNPs in the cytokine genes were included in GBM cases and controls. A total of 374 participants, including 72 GBM cases (44 males, 28 females; mean age at diagnosis 44 ± 15) and 302 controls (119 males, 183 females; mean age 46 ± 18) were successfully genotyped for further analysis. All of the tested tSNPs are in Hardy-Weinberg equilibrium (HWE) in the controls of this study (Table 1). The average tSNPs call rate was 99.12% in both cases and controls. χ^2^ test revealed one tSNP was significantly associated with GBM risk at a 5% level (rs1801275, *IL4R*, OR = 1.71, 95% CI, 1.00 - 2.92, *p* = 0.047).

**Table 1:** Basic information of candidate tSNP in this study

**SNP ID**	**Gene name**	**Chromosome position**	**Base change**	**MAF - Case**	**MAF - Control**	***p*-HWE**	**OR**	**95% CI**		***P***
rs1800871	IL10	1q32.1	T/C	0.340	0.332	0.095	1.04	0.71	1.52	0.854
rs568408	IL12A	3q25.33	G/A	0.160	0.159	0.888	1.00	0.61	1.64	0.994
rs20541	IL13	5q31.1	C/T	0.285	0.336	0.970	0.79	0.53	1.17	0.237
rs2070874	IL4	5q31.1	T/C	0.201	0.215	0.851	1.09	0.69	1.71	0.715
rs2243248	IL4	5q31.1	T/G	0.056	0.065	0.520	0.85	0.39	1.87	0.689
rs1801275	IL4R	16p12.1	A/G	0.125	0.196	0.181	1.71	1.00	2.92	0.047*
rs2069812	IL5	5q31.1	T/C	0.313	0.348	0.951	0.85	0.58	1.26	0.422

MAF: minor allele frequency; OR: odds ratio; 95% CI: 95% confidence interval.

* *p* < 0.05 indicates statistical significance.

We assumed that the minor allele of each tSNP was a risk factor compared to the wild-type allele. MAF of cases and controls are listed in Table 1. Genetic models were applied for analyzing the association between tSNPs and GBM risk by unconditional logistic regression analysis, which adjusted for age and gender. Our results showed that the genotype “TC” of rs20541 in *IL13* gene was associated with an increased risk of GBM in over-dominant model (OR = 2.00, 95% CI, 1.13- 3.54, *p* = 0.015). The genotype “CT” of rs1800871 in *IL10* gene showed a decrease risk in the over-dominant model (OR = 0.57, 95% CI, 0.33 – 0.97, *p* = 0.037). The genotype “AG” of rs1801275 in the *IL4R* gene showed an increase risk in the over-dominant model (OR = 2.29, 95% CI, 1.20 – 4.35, *p* = 0.0081), (Tables 2, 3 and 4).

**Table 2:** Single-SNP analysis

**Model**	**Genotype**	**Status = Case**	**Status = Control**	**OR (95% CI)**	***p*-value**	**AIC**	**BIC**
		**(N, %)**	**(N, %)**				
Co-dominant	C/C	41 (56.9%)	134 (44.4%)	1.00	0.051	353.0	372.6
	T/C	21 (29.2%)	133 (44.0%)	2.00 (1.11-3.62)			
	T/T	10 (13.9%)	35 (11.6%)	1.01 (0.45-2.27)			
Dominant	C/C	41 (56.9%)	134 (44.4%)	1.00	0.056	353.3	369.0
	T/C-T/T	31 (43.1%)	168 (55.6%)	1.68 (0.99-2.86)			
Recessive	C/C-T/C	62 (86.1%)	267 (88.4%)	1.00	0.480	356.5	372.2
	T/T	10 (13.9%)	35 (11.6%)	0.75 (0.35-1.64)			
Over-dominant	C/C-T/T	51 (70.8%)	169 (56.0%)	1.00	0.015*	351.0	366.7
	T/C	21 (29.2%)	133 (44.0%)	2.00 (1.13-3.54)			
Log-additive				1.24 (0.83-1.86)	0.280	355.8	371.5

SNP: rs20541 Percentage of typed samples: 374/374 (100%).

rs20541 association with response status (n = 374, adjusted by sex + age).

* *p* < 0.05 indicates statistical significance.

**Table 3:** Single-SNP analysis

**Model**	**Genotype**	**Status = Case**	**Status = Control**	**OR (95% CI)**	***p*-value**	**AIC**	**BIC**
		**(N, %)**	**(N, %)**				
Co-dominant	T/T	29 (40.3%)	141 (47.3%)	1.00	0.10	353.1	372.7
	C/T	37 (51.4%)	116 (38.9%)	0.59 (0.34-1.03)			
	C/C	6 (8.3%)	41 (13.8%)	1.23 (0.47-3.23)			
Dominant	T/T	29 (40.3%)	141 (47.3%)	1.00	0.16	353.7	369.3
	C/T-C/C	43 (59.7%)	157 (52.7%)	0.68 (0.40-1.17)			
Recessive	T/T-C/T	66 (91.7%)	257 (86.2%)	1.00	0.29	354.5	370.2
	C/C	6 (8.3%)	41 (13.8%)	1.61 (0.64-4.01)			
Over-dominant	T/T-C/C	35 (48.6%)	182 (61.1%)	1.00	0.037*	351.3	366.9
	C/T	37 (51.4%)	116 (38.9%)	0.57 (0.33-0.97)			
Log-additive				0.90 (0.61-1.32)	0.59	355.4	371.0

SNP: rs1800871 Percentage of typed sample: 370/374 (98.93%).

rs1800871 association with response status (n = 370, adjusted by sex + age).

* *p* < 0.05 indicates statistical significance.

**Table 4:** Single-SNP analysis

**Model**	**Genotype**	**Status = Case**	**Status = Control**	**OR (95% CI)**	***p*-value**	**AIC**	**BIC**
		**(N, %)**	**(N ,%)**				
Co-dominant	A/A	56 (77.8%)	187 (62.8%)	1.00	0.028	350.1	369.6
	A/G	14 (19.4%)	105 (35.2%)	2.26 (1.19-4.32)			
	G/G	2 (2.8%)	6 (2.0%)	0.72 (0.13-4.02)			
Dominant	A/A	56 (77.8%)	187 (62.8%)	1.00	0.016	349.4	365.1
	A/G-G/G	16 (22.2%)	111 (37.2%)	2.07 (1.12-3.83)			
Recessive	A/A-A/G	70 (97.2%)	292 (98.0%)	1.00	0.550	354.9	370.5
	G/G	2 (2.8%)	6 (2.0%)	0.58 (0.10-3.23)			
Over-dominant	A/A-G/G	58 (80.6%)	193 (64.8%)	1.00	0.0081*	348.2	363.9
	A/G	14 (19.4%)	105 (35.2%)	2.29 (1.20-4.35)			
Log-additive				1.76 (1.00-3.10)	0.042	351.1	366.7

SNP: rs1801275 Percentage of typed samples: 370/374 (98.93%).

rs1801275 association with response status (n = 370, adjusted by sex + age).

Note: AIC: Akaike Information Criterion; BIC: Bayesian Information Criterion.

* *p* < 0.05 indicates statistical significance.

We further analyzed whether the seven tSNPs have a different effect on GBM risk in gender specific population, and found that the allele “G” of rs2243248 in the *IL-4* gene showed a decrease risk in female (OR = 0.35, 95% CI,0.13 - 0.94, *p* = 0.0032), but the allele “T” showed a decrease risk in male (OR = 0.30, 95% CI, 0.17 - 0.53, *p* = 0.0032) (Table 5).

**Table 5:** Association between sex and the risk of GBM in the rs2243248

	**Female**			**Male**		
	**Status = Case**	**Status = Control**	**OR (95% CI)**	**Status = Case**	**Status = Control**	**OR (95% CI)**
T/T	21	160	1.00	43	103	0.30 (0.17-0.53)
G/T	7	23	0.35 (0.13-0.94)	1	16	1.78 (0.22-14.26)

rs2243248 and sex cross-classification interaction table (n = 374, adjusted by age).

Interaction *p*-value: 0.0032.

## Discussion

We genotyped seven tSNPs in this case–control study in the Han Chinese population, and found two risk tSNPs and one protective tSNP using genetic model analysis. In addition, we also found one tSNP have different risk effect on GBM in gender specific population. All the results suggested that the polymorphisms of these cytokine genes may play an important role in the risk of GBM in the Han Chinese population.

Interleukins are a part of cytokine, encoded by interleukin genes and produced by a variety of cells. They can deliver information, activate and regulate immune cells, mediate T and B cells activation, proliferation and differentiation. Cytokines play a significant role in cancer diagnosis, prognosis and therapy. The immune system’s failure to recognize the malignant tumor cells and perform an effective response may be the result of tumor-associated cytokine deregulation [15].

IL10 (interleukin-10) has pleiotropic effect in immunoregulation and inflammation, which plays a key role in immunosuppressive and antiangiogenic process, suggesting its possible involvement in carcinogenesis [16]. It has been demonstrated that polymorphisms of the *IL-10* gene are associated with multiple cancer, such as gastric cancer, non-small cell lung cancer and breast cancer [3,17-20], and our results indicating that the polymorphisms of *IL-10* are associated with GBM.

*IL13* encodes IL-13, an immunoregulatory cytokine produced primarily by activated Th2 cells. IL-13 is thought to the pathogenesis of allergen-induced asthma [21]. Besides, the polymorphisms of *IL-13* involved in some other diseases, such as eczema, allergic rhinitis [22,23]. GBM etiology remains unclear, but IL-13 has been shown to be over expressed in a majority of glioma cell lines and GBM tumor tissues [15]. There are consistent reports of inverse association between risk of adult glioma and personal history of allergy and autoimmune disease, but the molecular mechanism still unclear, there still need further investigate.

IL-4 is a ligand for interleukin-4 receptor, inducing macrophage activation and synergizing with colony-stimulating factors in promoting the growth of hematopoietic cells. Previously researches have suggested the *IL-4* polymorphisms were significantly associated with the risk of adult glioma [4]. Another report showed that IL-4 induced an aberrant activation of Stat3 in GBM cells but not in normal human astrocytes, and speculated that IL-4 induce aberrant activation of Stat3 may contribute to the pathogenesis of GBM cells [8].

*IL4R* encodes the alpha chain of the interleukin-4 receptor that can bind interleukin 4 and interleukin 13 to regulate IgE production [24]. A soluble form of the encoded protein can inhibit IL-4 mediated cell proliferation. In our study rs1801275 in the *IL4R* gene can predict 2.29-fold GBM susceptibility by the over-dominant model. In addition, another article also reported rs1801275 could increase the risk of GBM (OR = 1.61, 95% CI, 1.05 – 2.47) in a population-based case–control study [25], it is consistent with our results that *IL-4* gene are associated with GBM.

Helper T (Th) cell can secret multiple cytokines. Th1 cell mainly produced IL-2, IFN-γ and TNF, mediating cellular immune response and involving in delayed type hypersensitivity [26]. Th2 cell secreted *IL-4*, *IL-6*, *IL-10* and *IL-13*, mainly mediating humoral immune response [26]. Under normal circumstance, Th1 and Th2 cytokines are in dynamic equilibrium. In the anti-tumor immunity Th1 cytokines should have a more important role. But in glioma tissues, there is obviously predominant expression of Th2 type cytokines, it may result tumor cells escape from immune response, so the abnormal secretion of *IL-4*, *IL-10* and *IL-13* may play an important role in the occurring and developing of human glioma [27,28]. Besides, previously results showed that *IL4*, *IL4R* and *IL13* genes may play an important role in glioma survival [29]. All of these suggest multiple cytokines are associated with tumor development and progression survival.

In addition, gender difference should be considered in the association analysis, because many genes have been demonstrated function differently in male and female. Such as *5-HTTLPR* gene, females with the l/s genotype showed higher anxiety than those with the s/s genotype in both state and trait anxiety. Oppositely, males with the s/s genotype showed high anxiety than those with the l/s genotype [30]. The human gene *BDNF* genotyping 196G/G carriers can increase the risk of multiple sclerosis only in females, but not in males [31]. However, few researches take gender difference into consideration in association analysis of susceptibility gene. We analyzed whether cytokine genes have different effect on GBM in gender specific population, and found that the allele “G” of rs2243248 in the *IL-4* gene showed a decrease risk of GBM in female (OR = 0.35, 95% CI, 0.13 – 0.94, *p* = 0.0032), but the allele “T” showed a decrease risk in male (OR = 0.30, 95% CI, 0.17 – 0.53, *p* = 0.0032). We speculated that the expression of *IL-4* polymorphisms maybe regulated by sex hormone.

## Conclusions

In conclusion, our findings combined with previously results, suggest that the polymorphisms of cytokine genes have potential role in GBM development, and we advocate that gender difference should be taken into consideration in research of susceptible gene. However, the exact function of the polymorphisms of the genes and the regulatory mechanism for gene expression have not been researched clearly, the molecular mechanisms still need to be further investigated.

## Abbreviations

GBM: Glioblastoma; tSNP: Tag single nucleotide polymorphism; OR: Odd ratio; 95% CI: 95% confidence interval.

## Competing interests

The authors declare that they have no competing interests.

## Authors’ contributions

TBJ: conceived in the design of study, and performed the data management. XLL: participated in the design of study, and draft the manuscript. JYZ: participated in the design of study and helped to draft the manuscript. HW: designed the primers and carried out the genetic study. TTG: carried out the genetic study. GL: collected the blood samples and participated in the design of study. GDG: collected the blood samples, and participated in the design of study. CC: conceived in the design of the study. All authors read and approved the final manuscript.

## Pre-publication history

The pre-publication history for this paper can be accessed here:

http://www.biomedcentral.com/1471-2407/13/236/prepub
